# Autistic Traits, Arousal, and Gender Features in a Nonclinical Sample of Italian Adolescents

**DOI:** 10.3390/ijerph20010693

**Published:** 2022-12-30

**Authors:** Fiammetta Iannuzzo, Giovanni Genovese, Clara Lombardo, Carmenrita Infortuna, Rosa De Stefano, Carmela Mento, Maria Rosaria Anna Muscatello, Antonio Bruno

**Affiliations:** 1 Department of Biomedical and Dental Sciences and Morphofunctional Imaging, University of Messina, Via Consolare Valeria 1, Contesse, 98125 Messina, Italy; 2 Psychiatry Unit, Polyclinic Hospital University of Messina, Via Consolare Valeria 1, Contesse, 98125 Messina, Italy

**Keywords:** autism, autism spectrum disorder, adolescents, developmental disorders, broader autism phenotype

## Abstract

(1) Background: Subthreshold autism is a sub-clinical pattern of autism spectrum disorder-like (ASD-like) traits, including poor social skills, cognitive rigidity, anxiety, and aloofness. These ASD-like traits are significantly more prevalent among parents and relatives of participants with autism; however, evidence suggests that subclinical autistic traits are not restricted to the family members of individuals with autism but rather are continuously distributed in the general population. Though the autistic subclinical form is perhaps prevalent among adults, little attention has been paid to the association between autistic traits and global functioning in adolescence. The aim of the present study is to investigate the subthreshold autism phenotype in adolescence and its relationship with arousal correlates, exploring gender differences emerging in the sample. (2) Methods: A sample of 725 students (293 males and 432 females; mean age 17.19) were recruited from three high schools in Southern Italy. They were assessed by the following instruments: Autism Spectrum Quotient, Adult Autism Subthreshold Spectrum, Ritvo Autism and Asperger Diagnostic Scale 14, and Hyperarousal Scale. (3) Results: In males, significant direct correlations between all dimensions of arousal and all variables related to autistic traits emerged except for the correlations between the H-Scale “Introspection score”, the AQ questionnaire “Total score” (*p* = 0.094), and the AdAS-Spectrum questionnaire “Empathy factor” (*p* = 0.210); in females, significant positive correlations between all dimensions of arousal and all variables related to autistic traits emerged. (4) Conclusions: In the sample of adolescents with subclinical profiles of autistic traits, the Empathy factor of the AdAS Spectrum questionnaire was significantly higher in the male group than in the female group, underscoring lower empathic abilities in the former group. In the male group, the empathy factor did not have a statistically significant correlation with the H-scale introspection factor or with the autistic traits measured by AQ. We suppose that in male adolescents, another hypothetical factor seems to intervene in the relationship between autistic traits and arousal. Otherwise, empathy is a preponderant factor closely related to hyper-arousal responses in female adolescents with autistic traits.

## 1. Introduction

### 1.1. Autism Spectrum Disorder

Autism spectrum disorder (ASD) is a term used to describe a constellation of social communication and interaction deficits with the presence of unusual sensory motors and repetitive behaviours [1]. Since there are no available biomarkers for ASD, the diagnosis is possible based on behaviour. The Diagnostic and Statistical Manual of Mental Disorders (DSM-5) simplified the diagnosis, clarifying that the ASD spectrum is based on two domains: social communication and restricted, repetitive, or unusual sensory–motor behaviours [1]. This new spectrum includes diagnoses of subtypes of autism, such as Asperger’s disorder and pervasive developmental disorder [2]. The aetiology of autism seems to derive from genetic predispositions combined with environmental factors [3]; about 400–1000 genes are involved in susceptibility to autism [4]. Family studies estimated the heritability of ASD to be about 80%, meaning that the variation in ASD events in the population is due to inherited genetics, and twin studies assess the size of the phenotype variance due to genetic factors influences [5]. Although the possibility that environmental or non-genetic effects influence ASD is not excluded, few studies support this hypothesis [6]. ASD is associated with the deregulation of the autonomic nervous system (ANS) in terms of sympathetic hyper-arousal, parasympathetic undertone, and atypical interaction of the two systems [7]. In subjects with autism, hypo-reactivity (lack of reaction to stimuli) and hyper-reactivity (excess of reaction to stimuli) have long been observed [8]. The prevalence of abnormal behavioural responses to stimuli among subjects with autism led scientific research to study physiological reactivity in this population. The physiological reactivity (PR), indicated as arousal, can be over-expressed or hypo-expressed in subjects with ASD that often experience abnormalities in sensation and attention [9]. These abnormalities in PR are a risk factor for atypical behaviour and psychiatric diseases in autism. While hyper-arousal seems to be associated with fear, anxiety symptoms, and avoidance, hypo-arousal is associated with obtuseness and under-stimulation [10].

Additionally, low levels of PR are linked to externalizing symptoms, such as violent behaviour, while high levels of PR are associated with internalizing symptoms, such as anxiety [11]. Activation of the ANS in response to external inducement or stress is commonly measured through heart rate (HR), heart rate variability (HRV), blood pressure (BP), and electrodermal activity (EDA) [12]. In many studies, these vital signs showed deficits in arousal regulation in ASD [13,14]. The discrepancies in the existing literature on arousal features in ASD suggest large heterogeneity in this population, as well as a central role of arousal in the relationship between autism and psychiatric symptoms. Due to the large aetiological heterogeneity associated with ASD [15], this disorder shows significant variability in symptoms in clinical populations. In childhood and adolescence, there are specific patterns of disease, including atypical response to social stimuli linked to social and communication impairment (i.e., gaze detection, attention to gaze, eye tracking), and atypical control of physiologic mechanisms linked to emotional, cognitive, and social development (i.e., sleep-wake cycle, the autonomic nervous system, and visceral sensation) [13,16,17]. 

Clinical ASD diagnosis in adulthood is lower than in that of children; this is maybe because people with the clearest ASD symptoms have already been diagnosed [18]. Regarding gender differences, ASD seems to be more frequent in males than in females [19], and some theories have explained this gender distribution. One of these theories is that the male sex tends towards better systematisation rather than empathisation, and for this reason, autism is considered to be an extreme of a normal male cognitive profile [20]. In addition, there is the possibility that women are underdiagnosed or diagnosed later because ASD is masked by female gender-related social differences [21]. 

### 1.2. Sub-Clinical Form of Autism Spectrum Disorder

Rather than a full-blown clinical disorder, the most common phenotypic presentation of ASD seems to be characterized by subclinical autistic traits. These subclinical traits have been defined as broader autism phenotype (BAP) [22,23]. BAP is a term indicating the presence of sub-clinical ASD patterns, including poor social skills, cognitive rigidity, anxiety, and aloofness. These ASD-like traits are significantly more prevalent among the parents and relatives of subjects with autism; however, evidence suggests that subclinical autistic traits are not restricted to family members of individuals with autism but rather are continuously distributed in the general population [24,25]. The autistic subclinical form is perhaps prevalent among adults. Indeed, the course of subclinical autistic forms seems to be frequently complicated by other mental disorders, including anxiety, mood disorders, psychotic disorders, trauma, and stressor-related disorders. For these reasons, adult patients receive clinical attention at the onset of these disorders, and their autistic features may remain underdiagnosed. 

Autistic subthreshold traits in adults concern difficulties with integrating into groups and building relationships with and avoidance of people. These people have restricted and specific interests and used to have solipsistic dialogues and imaginary companions. They could find it easier to establish a relationship with younger or older individuals or spend time with pets rather than with humans. For them, the internet may be their favourite communication instrument. They may not understand social interactions, and they often show paradoxical empathy; therefore, they may develop compensatory mechanisms, such as emulating and imitating others [26]. While BAP was detected among adults, especially among parents of ASD probands, little attention has been paid to the association between autistic traits (AT) and global functioning in adolescence. Adolescence is characterized by dramatic changes in terms of behaviour and neuroplasticity due to the presence of “critical periods”. These periods are windows of susceptibility to stressors resulting in a greater alteration of brain developmental trajectories that lead to a major vulnerability to mental illness [27]. During this critical period, sex differences could provide a critically important direction for explaining the aetiology of various illnesses that show large sex differences in prevalence [28].

Moving from these assumptions, the first aim of the present study was to investigate the subthreshold autism phenotype in adolescence since few studies have dealt with this critical age. Moreover, this study aimed to investigate the relationships between arousal and autism traits in a significant cohort of adolescents. In addition, gender differences in brain development and maturation processes should be considered essential to understanding the different predispositions of males and females to develop mental disorders. For this reason, the present study also sought to explore if there are gender differences in the association between arousal features and ASD traits in an adolescent sample.

## 2. Materials and Methods

### 2.1. Procedure

The study was developed for high-school students (first- to fifth-year classes) after authorisation and consent were obtained from school departments. Data were collected from a sample of 752 students recruited from three high schools (economic technical institute, scientific high school, and classical high school) in two cities from Southern Italy that have agreed to participate through surveys distributed in classrooms. All subjects and their parents gave written consent to participate in this study. Minors were asked to take home a letter to their parents explaining the aim of the research. Parents were assured that students’ responses would be treated as confidential. Students who agreed to participate and those who received parental permission were involved in a single group testing session (up to 60 min) under the coordination of two experienced psychiatrists. Teachers remained in the classroom to supervise student behaviour. Before the administration of the survey, the study was reviewed and approved by the participating school districts. Data collection covered five months.

### 2.2. Measures

All participants were assessed using the following instruments:

(1) Autism Spectrum Quotient (AQ) [29]: a self-administered questionnaire comprised of 50 items. It consisted of five subscales, each of 10 questions assessing: Social Skills, Communication, Imagination, Attention to Detail, and Attention–Switching. Participants were asked to answer each question by circling their response on a 4-point scale (totally agree, partially agree, partially disagree, totally disagree). A total AQ score was calculated by summing all the scores for each item, with a maximum score of 200. Autistic traits were considered potentially clinically significant when a total score greater than or equal to 32 was reported. The Autism-Spectrum Quotient has had both good internal consistency (α ≥ 0.70) and test–retest reliability [30].
(2) Adult Autism Subthreshold Spectrum (AdAS Spectrum) [31]: The questionnaire included 160 items exploring the wide spectrum in the manifestation of autism organized into seven domains. The childhood/adolescence domain described symptoms occurring during these ages. The verbal communication domain described difficulties or impairment in speaking to someone or in intervening in a conversation. The non-verbal communication domain explored difficulty in looking at others, hugging, kissing, or holding someone by the hand, for example. The empathy domain explored impairment in emphatic abilities. The inflexibility and adherence to routine domain explored rigidity traits regarding work or relationships with others. The restricted interests and rumination domain encompassed these two typical autistic symptoms. The hyper-hypo reactivity to sensory input explored in great detail the tendency to over- or under-react to physical stimuli. Item responses were coded in a dichotomous way (yes/no), and domain scores were obtained by counting the number of positive answers. Internal consistency for the AdAS Spectrum total score has been excellent (Kuder-Richardson’s coefficient = 0.96). Five domains have proven good internal consistency (α ≥ 0.80) [32].
(3) Ritvo Autism and Asperger Diagnostic Scale 14 (RAADS-14) [33]: screening tool consisting of 14 questions assessing autistic symptoms. The scale derived from the Ritvo Autism and Asperger Diagnostic Scale Revised (RAADS-R), which assessed the presence of symptoms attributable to Asperger’s Disorder and mild DSA. The response alternatives to each statement were given on a four-point Likert scale (ranging from 0 to 3), indicating the duration of each symptom. A factor analysis identified three factors consistent with mentalizing deficits, social anxiety, and sensory reactivity relevant to the diagnosis of ASD. RAADS-R has shown good internal consistencies (α > 0.8) for three domains and poor consistency (α = 0.42) for the language domain [34].
(4) Hyperarousal Scale (H-Scale) [35,36]: A self-assessment scale that consisted of 26 items that measured the behavioural trait of hyperarousal on a four-point Likert scale (from 0 to 3, where 0 = not at all, 1 = a little, 2 = a lot, and 3 = very much). The scale provided a Total Score (HSUM): the higher the total (max. 78), the greater the level of hyperarousal. A cut-off ≥ 45 was indicative of pathological levels of hyperarousal. Three subscales were also used: “Introspectivity”, indicating a possible tendency to ruminate, included six items (4; 5; 9; 11; 22; 23) and provided a scoring range of 0–18; “Reactivity”, indicative of “Alert response”, included three items (6; 12; 17) with a score range of 0–9; “Extreme Responses” referred to the total number of “Very Many” responses and included a score range of 0–26. The Internal consistency of the H-scale was adequate (α= 0.81) [35].

### 2.3. Statistical Analysis

All statistical analyses were carried out using the SPSS 25.0 version. Continuous data were expressed as mean ± standard deviation, and differences between males and females were assessed by Student’s *t*-test for independent samples. A correlation analysis (Pearson correlation), controlled for gender and age, was performed on the total sample to assess possible associations between arousal dimensions and autistic traits. Subsequently, in order to evaluate gender differences, two correlation analyses were performed separately in males and females. Results were considered significant for *p* values < 0.05. Additionally, a Bonferroni correction was performed for the number of tests in each group (*p* ≤ 0.003).

## 3. Results

Of the total sample (752 students), 21 subjects (8 males and 13 females) were excluded because their questionnaires were incomplete, and 6 subjects (0.8%) were excluded because they had clinically significant autistic traits as measured by the Autism Spectrum Quotient—AQ questionnaire. The final 725 subjects enrolled (40.4% males, 59.6% females) were characterized by a mean age of 17.19 ± 0.78 years (age range 16–18 years); no significant differences in age between males and females emerged (Mean age ± S.D. Males vs. Females: 17.19 ± 0.81 vs. 17.20 ± 0.77; *p* = 0.943).

Table 1 reports descriptive statistics and gender differences related to the diagnostic instruments administered. Statistical analysis revealed significant differences in the AQ questionnaire “Total score” (*p* = 0.021) and in the “Childhood/Adolescence” (*p* = 0.022) and “Empathy” (*p* = 0.015) AdAS-Spectrum factors, which were higher in males, and in “Sensory Reactivity” (*p* = <0.0001) RAADS14 factor, “I score” (*p* = 0.004), and “Total score” (*p* = 0.016) H-Scale variables, which were higher in females.

Table 2 shows the correlation analysis (controlled for gender and age) carried out to evaluate the possible associations between dimensions of arousal, measured by the H-Scale questionnaire, and autistic traits assessed by AQ, AdAS-Spectrum, and RAADS14 questionnaires. The analysis revealed significant direct correlations between all dimensions of arousal and all variables related to autistic traits. After the Bonferroni correction was performed, the results remained unchanged.

Table 3 and Table 4 show the correlation analyses (Pearson correlation) between arousal (H-Scale) and autistic traits (AQ, AdAS-Spectrum, and RAADS14) performed separately in males and females, respectively, in order to evaluate gender differences.

In males, from data analyses, significant direct correlations between all dimensions of arousal and all variables related to autistic traits emerged, except for the correlations between the H-Scale “Introspection score”, the AQ questionnaire “Total score” (*p* = 0.094), and the AdAS-Spectrum questionnaire “Empathy factor” (*p* = 0.210). Moreover, after the Bonferroni correction was performed, the direct correlation between the H-Scale “Introspection score” and the AdAS-Spectrum questionnaire “Social Anxiety” became non-significant (Table 3).

In females, statistical analysis showed significant positive correlations between all dimensions of arousal and all variables related to autistic traits. When the Bonferroni correction was performed, the results remained unchanged, except for the relationship between the H-Scale “Extreme” score and the AdAS-Spectrum questionnaire “Empathy” dimension (Table 4).

## 4. Discussion

To our knowledge, this is the first study to investigate subthreshold autism in a cohort of adolescents and its relationships with arousal. Most research has focused on the correlates of autism spectrum disorders in childhood or adulthood, excluding adolescence. One of our results indicated a correlation between all measured autism spectrum variables and all dimensions of arousal in the total sample. This finding is similar to other studies that explored the correlation between autistic traits and arousal levels in children [9,37].

However, the current study found gender differences among the students. The empathy factor of the AdAS Spectrum questionnaire was significantly higher in the male group than in the female group, underscoring lower empathic abilities in the former group. In the male group, the empathy factor did not have a statistically significant correlation with the H-scale introspection factor or with the autistic traits measured by AQ. On the contrary, these correlations were predominant in the female group.

The differences found in empathy levels between females and males agree with the current literature. Empathy is an essential component of human social life, and it consists of the ability to understand another’s mental state and respond with an appropriate emotion or action [38]. Empathy has two components: cognitive empathy, namely the ability to understand another person’s emotive status, and emotional empathy, namely the observer’s emotional response to the mental state of others [39]. Studies have shown that individuals with ASD have deficits in emphatic responses [40]. Although Baron-Cohen et collaborators did not find typical sex differences among adults with ASD in a 2011 study that measured cognitive empathy, behavioural sex differences emerged between males and females with ASD (i.e., sensory symptoms, socio-communication deficits) [41].

Some authors have hypothesized that the difficulty in diagnosing ASD in the female sex might be due to camouflaging in response to social demands [42]. Camouflage techniques are coping strategies used to disguise difficulties during social situations; examples of camouflaging include making eye contact during a conversation, using pre-prepared jokes or phrases during a conversation, imitating other’s social behaviour, facial expressions, gestures, and learning social dialogues [43]. Evidence shows that camouflaging may play a role in male-preponderance autism prevalence. Indeed, females are more likely or motivated to camouflage, and thereby, they go undetected and undiagnosed [43,44]. Our results show a greater correlation between empathy, autistic traits, and arousal in the female group. This finding suggests that autistic traits, supported by higher autonomic reactivity, might remain subthreshold in women due to a stronger empathic ability, perhaps the basis of the camouflage. Allemand et al. [45], in a prospective study, explored the predictive associations between empathy development in adolescence and self-reported social competencies in adulthood. They found a general increase in empathy levels during adolescence and interindividual differences in level and change of empathy in adolescents. Furthermore, they showed that the female group presented higher levels of empathy than the male group, although the two groups did not show different empathy development across the adolescent years. According to Allemand et al., developmental trajectories in empathy do not differ in the two genders, but our results show new findings, suggesting that only the female sex shows a significant correlation between empathy, the presence of autistic traits, and arousal levels.

Normal levels of arousal are an important aspect of personal, empathic response. However, higher or lower levels of arousal could compromise behavioural flexibility and prosocial behaviours [3]. The females’ group had significantly higher levels at the sensory reactivity factor of the RAADS14 and the total score of the H-scale, rather than the male group. This result indicates a great gender difference in arousal levels and that females are more likely to respond with hyper-arousal reactions to empathic situations. Bons et al. [46], in a review of the motor, emotional, and cognitive aspects of empathy in children and adolescents with ASD and Conduct Disorders (CD), hypothesized that the lack of attention to the eyes, a common substrate in the two pathologies, is responsible for the alteration of motor and cognitive components of empathy. On the contrary, the differences between ADS and CD in the emotional component of empathy could be related to changes in autonomic responsivity and the amygdala, closely related to levels of arousal. Specifically, they identified hyper-responsive emotional empathy in ASD and hypo-responsive emotional empathy in CD. Our results, despite a different measurement of arousal levels, show that this correlation may be valid in a healthy sample of female adolescents. We suppose that in male adolescents, another hypothetical factor seems to intervene in the relationship between empathy, arousal, and autistic traits. Giarelli et al. [47] underline the prevalence of externalizing experiences in males, and this observation could be considered a relevant variable in explaining the aforementioned data, although new studies are required.

Undoubtedly, this study had several limitations. Firstly, the research sample consisted of high school students taken from an urban area of Southern Italy. Results would have been more solid if the sample had been more heterogeneous regarding age and cultural/socioeconomic variables. Furthermore, the lack of evaluation of behavioural variables (e.g., drug use, alcohol use, presence of comorbidities, presence of internalizing and externalizing symptoms, and presence of family members diagnosed with autism) and the correlational study design do not allow for the exclusion of the possible effect of these features on the obtained results. Moreover, the cross-sectional nature of the data and its convenience-sampling strategy limited the scope and the potential impact of the study. Finally, we only applied self-reported instruments, and the Italian version of the H-scale has not been validated on adolescents’ samples; however, the sample recruited in the study had a mean age of 17.19 years with low variability (S.D. = 0.78) and allowed us to consider it to be very close (and similar) to the sample used for the standardisation of the instrument and be fairly certain of the validity of the achieved results.

## 5. Conclusions

Despite the limitations, the main result of this study suggests empathy as a preponderant factor closely related to hyper-arousal responses in female adolescents with autistic traits. Moreover, we can take into consideration the empathic factor as responsible for camouflage techniques. Since camouflaging is typical in female subjects with ASD, we suppose that it is even present in female subthreshold autism, perhaps enhanced by empathic abilities. Further studies are necessary to explore the nature of empathic abilities in subthreshold autism, clarifying the differences between the overt forms of ASD and among the two genders. In addition, the hyper-reactivity of the female sample as a reaction to empathic skill is a result that needs to be investigated.

## Figures and Tables

**Table 1:** Descriptive statistics and gender differences on psychological tests administered.

	Total Sample (n = 725)		Males (n = 293)		Females (n = 432)		Student Test
	Media	S.D.	Mean	S.D.	Mean	S.D.	*p*
AQ	17.45	5.159	17.99	5.346	17.09	5.001	0.021
AdAS-Spectrum							
Childhood/adolescence	6.82	3.375	7.17	3.642	6.58	3.164	0.022
Verbal communication	6.17	2.853	5.98	3.007	6.29	2.740	0.150
Non verbal communication	10.37	4.295	10.56	4.547	10.24	4.116	0.315
Empathy	3.41	2.372	3.67	2.554	3.24	2.226	0.015
Inflexibility	16.61	6.500	16.40	7.178	16.75	6.001	0.466
Restricted intresting and rumination	9.38	4.165	9.22	4.462	9.50	3.953	0.376
Reactivity to stimuli	5.46	3.320	5.56	3.532	5.40	3.170	0.515
Total score	75.59	23.856	76.33	25.922	75.08	22.369	0.490
RAADS14							
Mentalizing deficit	5.75	4.421	5.89	4.861	5.65	4.099	0.469
Social anxiety	2.51	2.731	2.65	2.795	2.41	2.686	0.242
Sensory reactivity	3.40	2.558	2.98	2.430	3.68	2.605	<0.0001
Total score	11.62	7.758	11.51	8.504	11.69	7.218	0.768
H SCALE							
I score	10.54	2.892	10.16	2.917	10.80	2.850	0.004
R score	3.33	1.924	3.18	2.044	3.42	1.834	0.104
Extreme	3.93	3.851	3.69	4.071	4.09	3.691	0.168
Total score	37.63	9.448	36.61	10.432	38.33	8.661	0.016

**Table 2:** Correlation analysis (Pearson correlation) controlled for gender and age between arousal (H-Scale) and autistic traits (AQ, AdAS-Spectrum and RAADS14) in total sample.

	AQ	AdAS-Spectrum								RAADS14			
H SCALE	Total Score	Childhood/Adulthood	Verbal Communication	Non Verbal Communication	Empathy	Inflexibility	Restricted Intresting and Rumination	Reactivity to Stimuli	Total Score	Mentalizingn Deficits	Social Anxiety	Sensory Reactivity	Total Score
I Score	0.186 **	0.352 **	0.295 **	0.416 **	0.126 *	0.466 **	0.515 **	0.325 **	0.477 **	0.275 **	0.188 **	0.256 **	0.299 **
R Score	0.324 **	0.331 **	0.383 **	0.395 **	0.236 **	0.473 **	0.431 **	0.485 **	0.529 **	0.430 **	0.373 **	0.437 **	0.514 **
Extreme	0.259 **	0.342 **	0.299 **	0.372 **	0.182 **	0.412 **	0.438 **	0.361 **	0.468 **	0.319 **	0.291 **	0.298 **	0.374 **
Total SCORE	0.325 **	0.428 **	0.383 **	0.484 **	0.208 **	0.533 **	0.565 **	0.430 **	0.589 **	0.418 **	0.365 **	0.416 **	0.498 **

* *p* = 0.001; ** *p* < 0.0001.

**Table 3:** Correlation analysis (Pearson correlation) between arousal (H-Scale) and autistic traits (AQ, AdAS-Spectrum and RAADS14) in males.

	AQ	AdAS-Spectrum								RAADS14			
H SCALE	Total Score	Childhood/Adulthood	Verbal Communication	Non Verbal Communication	Empathy	Inflexibility	Restricted Intresting and Rumination	Reactivity to Stimuli	Total Score	Mentalizing Deficits	Social Anxiety	Sensory Reactivity	Total Score
I Score	0.098	0.293 **	0.279 **	0.365 **	0.073	0.406 **	0.431 **	0.218 **	0.386 **	0.242 **	0.161 *^§^	0.220 **	0.255 **
R Score	0.273 **	0.244 **	0.405 **	0.323 **	0.283 **	0.403 **	0.364 **	0.421 **	0.451 **	0.486 **	0.435	0.420 **	0.541 **
Extreme	0.203 **	0.258 **	0.270 **	0.307 **	0.264 **	0.368 **	0.363 **	0.290 **	0.402 **	0.369 **	0.296	0.280 **	0.388 **
Total score	0.269 **	0.335 **	0.393 **	0.394 **	0.248 **	0.463 **	0.469 **	0.337 **	0.496 **	0.450 **	0.373	0.369 **	0.486 **

* *p* = 0.006; ** *p* < 0.0001; ^§^ Non- significant after Bonferroni correction.

**Table 4:** Correlation analysis (Pearson correlation) between arousal (H-Scale) and autistic traits (AQ, AdAS-Spectrum and RAADS14) in females.

	AQ	AdAS-Spectrum								RAADS14			
H SCALE	Total Score	Childhood/Adulthood	Verbal Communication	Non Verbal Communication	Empathy	Inflexibility	Restricted Intresting and Rumination	Reactivity to Stimuli	Total Score	Mentalizing Deficits	Social Anxiety	Sensory Reactivity	Total Score
I Score	0.242 **	0.374 **	0.293 **	0.439 **	0.169 **	0.484 **	0.552 **	0.404 **	0.525 **	0.306 **	0.203 **	0.293 **	0.338 **
R Score	0.362 **	0.390 **	0.385 **	0.445 **	0.225 **	0.510 **	0.440 **	0.512 **	0.575 **	0.367 **	0.337 **	0.432 **	0.474 **
Extreme	0.303 **	0.380 **	0.317 **	0.413 **	0.135 *^§^	0.445 **	0.493 **	0.437 **	0.518 **	0.293 **	0.295 **	0.340 **	0.383 **
Totale	0.365 **	0.473 **	0.375 **	0.532 **	0.204 **	0.571 **	0.610 **	0.504 **	0.645 **	0.376 **	0.355 **	0.455 **	0.496 **

* *p* = 0.005; ** *p* < 0.0001; ^§^ Non- significant after Bonferroni correction.

## Data Availability

The data are kept by the authors who reserve the right to make them available for possible replications of the study.

## References

[1] American Psychiatric Association (2013). American Psychiatric Association Diagnostic and Statistical Manual of Mental Disorders: DSM-5.

[2] Lord C., Elsabbagh M., Baird G., Veenstra-Vanderweele J. (2018). Autism spectrum disorder. Lancet.

[3] Newschaffer C.J., Croen L.A., Daniels J., Giarelli E., Grether J.K., Levy S.E., Mandell D.S., Miller L.A., Pinto-Martin J., Reaven J. (2007). The Epidemiology of Autism Spectrum Disorders. Annu. Rev. Public Health.

[4] Ronemus M., Iossifov I., Levy D., Wigler M. (2014). The role of de novo mutations in the genetics of autism spectrum disorders. Nat. Rev. Genet..

[5] Bai D., Yip B.H.K., Windham G.C., Sourander A., Francis R., Yoffe R., Glasson E., Mahjani B., Suominen A., Leonard H. (2019). Association of Genetic and Environmental Factors With Autism in a 5-Country Cohort. JAMA Psychiatry.

[6] Tick B., Bolton P., Happé F., Rutter M., Rijsdijk F. (2016). Heritability of autism spectrum disorders: A meta-analysis of twin studies. J. Child Psychol. Psychiatry.

[7] Panju S., Brian J., Dupuis A., Anagnostou E., Kushki A. (2015). Atypical sympathetic arousal in children with autism spectrum disorder and its association with anxiety symptomatology. Mol. Autism.

[8] Liss M., Saulnier C., Fein D., Kinsbourne M. (2006). Sensory and attention abnormalities in autistic spectrum disorders. Autism.

[9] Rogers S.J., Ozonoff S. (2005). Annotation: What do we know about sensory dysfunction in autism? A critical review of the empirical evidence. J. Child Psychol. Psychiatry.

[10] Dalton K.M., Nacewicz B.M., Johnstone T., Schaefer H.S., Gernsbacher M.A., Goldsmith H.H., Alexander A.L., Davidson R.J. (2005). Gaze fixation and the neural circuitry of face processing in autism. Nat. Neurosci..

[11] Bauer A.M., Quas J.A., Boyce W.T. (2002). Associations between Physiological Reactivity and Children’s Behavior: Advantages of a Multisystem Approach. J. Dev. Behav. Pediatr..

[12] Ordaz S., Luna B. (2012). Sex differences in physiological reactivity to acute psychosocial stress in adolescence. Psychoneuroendocrinology.

[13] Dijkhuis R.R., Ziermans T., van Rijn S., Staal W., Swaab H. (2019). Emotional Arousal during Social Stress in Young Adults with Autism: Insights from Heart Rate, Heart Rate Variability and Self-Report. J. Autism Dev. Disord..

[14] Prince E.B., Kim E.S., Wall C.A., Gisin E., Goodwin M.S., Simmons E.S., Chawarska K., Shic F. (2017). The relationship between autism symptoms and arousal level in toddlers with autism spectrum disorder, as measured by electrodermal activity. Autism.

[15] An J.Y., Claudianos C. (2016). Genetic heterogeneity in autism: From single gene to a pathway perspective. Neurosci. Biobehav. Rev..

[16] Wang Q., Hoi S.P., Wang Y., Lam C.M., Fang F., Yi L. (2020). Gaze response to others’ gaze following in children with and without autism. J. Abnorm. Psychol..

[17] Sasson N.J., Touchstone E.W. (2014). Visual Attention to Competing Social and Object Images by Preschool Children with Autism Spectrum Disorder. J. Autism Dev. Disord..

[18] Happé F.G., Mansour H., Barrett P., Brown T., Abbott P., Charlton R.A. (2016). Demographic and Cognitive Profile of Individuals Seeking a Diagnosis of Autism Spectrum Disorder in Adulthood. J. Autism Dev. Disord..

[19] Masi A., DeMayo M.M., Glozier N., Guastella A.J. (2017). An Overview of Autism Spectrum Disorder, Heterogeneity and Treatment Options. Neurosci. Bull..

[20] Baron-Cohen S., Knickmeyer R.C., Belmonte M.K. (2005). Sex Differences in the Brain: Implications for Explaining Autism. Science.

[21] Halladay A.K., Bishop S., Constantino J.N., Daniels A.M., Koenig K., Palmer K., Messinger D., Pelphrey K., Sanders S.J., Singer A.T. (2015). Sex and gender differences in autism spectrum disorder: Summarizing evidence gaps and identifying emerging areas of priority. Mol. Autism.

[22] Sucksmith E., Roth I., Hoekstra R.A. (2011). Autistic Traits below the Clinical Threshold: Re-examining the Broader Autism Phenotype in the 21st Century. Neuropsychol. Rev..

[23] Bora E., Aydın A., Saraç T., Kadak M.T., Köse S. (2017). Heterogeneity of subclinical autistic traits among parents of children with autism spectrum disorder: Identifying the broader autism phenotype with a data-driven method. Autism Res..

[24] Wainer A.L., Ingersoll B.R., Hopwood C.J. (2011). The Structure and Nature of the Broader Autism Phenotype in a Non-clinical Sample. J. Psychopathol. Behav. Assess..

[25] Stewart M.E., Austin E.J. (2009). The structure of the Autism-Spectrum Quotient (AQ): Evidence from a student sample in Scotland. Pers. Individ. Dif..

[26] Dell’Osso L., Luche R.D., Gesi C., Moroni I., Carmassi C., Maj M. (2016). From Asperger’s Autistischen Psychopathen to DSM-5 Autism Spectrum Disorder and Beyond: A Subthreshold Autism Spectrum Model. Clin. Pract. Epidemiol. Ment. Health.

[27] Ismail F.Y., Fatemi A., Johnston M.V. (2017). Cerebral plasticity: Windows of opportunity in the developing brain. Eur. J. Paediatr. Neurol..

[28] Peper J.S., Burke S.M., Wierenga L.M. (2020). Sex differences and brain development during puberty and adolescence. Handb. Clin. Neurol..

[29] Baron-Cohen S., Wheelwright S., Skinner R., Martin J., Clubley E. (2001). The autism-spectrum quotient (AQ): Evidence from Asperger syndrome/high-functioning autism, males and females, scientists and mathematicians. J. Autism Dev. Disord..

[30] Stevenson J.L., Hart K.R. (2017). Psychometric Properties of the Autism-Spectrum Quotient for Assessing Low and High Levels of Autistic Traits in College Students. J. Autism Dev. Disord..

[31] Dell’Osso L., Gesi C., Massimetti E., Cremone I.M., Barbuti M., Maccariello G., Moroni I., Barlati S., Castellini G., Luciano M. (2017). Adult Autism Subthreshold Spectrum (AdAS Spectrum): Validation of a questionnaire investigating subthreshold autism spectrum. Compr. Psychiatry.

[32] Donati M.A., Berrocal C., Primi C., Petracchi G., Carpita B., Cosci F., Ruiz A., Carmassi C., Dell’Osso L. (2019). Measuring subthreshold autistic traits in the general population: Psychometric properties of the Adult Autism Subthreshold Spectrum (AdAS Spectrum) scale. Psychiatry Res..

[33] Eriksson J.M., Andersen L.M., Bejerot S. (2013). RAADS-14 Screen: Validity of a screening tool for autism spectrum disorder in an adult psychiatric population. Mol. Autism.

[34] Ritvo R.A., Ritvo E.R., Guthrie D., Ritvo M.J., Hufnagel D.H., McMahon W., Tonge B., Mataix-Cols D., Jassi A., Attwood T. (2011). The Ritvo Autism Asperger Diagnostic Scale-Revised (RAADS-R): A Scale to Assist the Diagnosis of Autism Spectrum Disorder in Adults: An International Validation Study. J. Autism Dev. Disord..

[35] Bruno A., Rizzo A., Muscatello M.R.A., Celebre L., Silvestri M.C., Zoccali R.A., Mento C. (2020). Hyperarousal Scale: Italian Cultural Validation, Age and Gender Differences in a Nonclinical Population. Int. J. Environ. Res. Public Health.

[36] Regestein QR D.J.H.M.M.B.P.M. (1993). Daytime alertness in patients with primary insomnia. Am. J. Psychiatry.

[37] Benevides T.W., Lane S.J. (2015). A Review of Cardiac Autonomic Measures: Considerations for Examination of Physiological Response in Children with Autism Spectrum Disorder. J. Autism Dev. Disord..

[38] Harmsen I.E. (2019). Empathy in Autism Spectrum Disorder. J. Autism Dev. Disord..

[39] Cox C.L., Uddin L.Q., Di Martino A., Castellanos F.X., Milham M.P., Kelly C. (2012). The balance between feeling and knowing: Affective and cognitive empathy are reflected in the brain’s intrinsic functional dynamics. Soc. Cogn. Affect. Neurosci..

[40] Baron-Cohen S., Wheelwright S. (2004). The Empathy Quotient: An Investigation of Adults with Asperger Syndrome or High Functioning Autism, and Normal Sex Differences. J. Autism Dev. Disord..

[41] Lai M.-C., Lombardo M.V., Pasco G., Ruigrok A.N.V., Wheelwright S.J., Sadek S.A., Chakrabarti B., Baron-Cohen S. (2011). A Behavioral Comparison of Male and Female Adults with High Functioning Autism Spectrum Conditions. PLoS ONE.

[42] Hull L., Petrides K.V., Allison C., Smith P., Baron-Cohen S., Lai M.-C., Mandy W. (2017). “Putting on My Best Normal”: Social Camouflaging in Adults with Autism Spectrum Conditions. J. Autism Dev. Disord..

[43] Lai M.-C., Lombardo M.V., Ruigrok A.N., Chakrabarti B., Auyeung B., Szatmari P., Happé F., Baron-Cohen S. (2017). Quantifying and exploring camouflaging in men and women with autism. Autism.

[44] Tierney S., Burns J., Kilbey E. (2016). Looking behind the mask: Social coping strategies of girls on the autistic spectrum. Res. Autism Spectr. Disord..

[45] Allemand M., Steiger A.E., Fend H.A. (2015). Empathy Development in Adolescence Predicts Social Competencies in Adulthood. J. Pers..

[46] Bons D., van den Broek E., Scheepers F., Herpers P., Rommelse N., Buitelaaar J.K. (2013). Motor, Emotional, and Cognitive Empathy in Children and Adolescents with Autism Spectrum Disorder and Conduct Disorder. J. Abnorm. Child Psychol..

[47] Giarelli E., Wiggins L.D., Rice C.E., Levy S.E., Kirby R.S., Pinto-Martin J., Mandell D. (2010). Sex differences in the evaluation and diagnosis of autism spectrum disorders among children. Disabil. Health J..

